# Chloride Secretion Induced by Rotavirus Is Oxidative Stress-Dependent and Inhibited by *Saccharomyces boulardii* in Human Enterocytes

**DOI:** 10.1371/journal.pone.0099830

**Published:** 2014-06-11

**Authors:** Vittoria Buccigrossi, Gabriella Laudiero, Carla Russo, Erasmo Miele, Morena Sofia, Marina Monini, Franco Maria Ruggeri, Alfredo Guarino

**Affiliations:** 1 Department of Translational Medical Science, Section of Pediatrics, University of Naples “Federico II”, Naples, Italy; 2 Department of Veterinary Public Health & Food Safety, Viral Zoonoses Unit, Istituto Superiore di Sanità Rome – Italy, Rome, Italy; University of North Carolina School of Medicine, United States of America

## Abstract

Rotavirus (RV) infection causes watery diarrhea via multiple mechanisms, primarily chloride secretion in intestinal epithelial cell. The chloride secretion largely depends on non-structural protein 4 (NSP4) enterotoxic activity in human enterocytes through mechanisms that have not been defined. Redox imbalance is a common event in cells infected by viruses, but the role of oxidative stress in RV infection is unknown. RV SA11 induced chloride secretion in association with an increase in reactive oxygen species (ROS) in Caco-2 cells. The ratio between reduced (GSH) and oxidized (GSSG) glutathione was decreased by RV. The same effects were observed when purified NSP4 was added to Caco-2 cells. N-acetylcysteine (NAC), a potent antioxidant, strongly inhibited the increase in ROS and GSH imbalance. These results suggest a link between oxidative stress and RV-induced diarrhea. Because *Saccharomyces boulardii* (Sb) has been effectively used to treat RV diarrhea, we tested its effects on RV-infected cells. Sb supernatant prevented RV-induced oxidative stress and strongly inhibited chloride secretion in Caco-2 cells. These results were confirmed in an organ culture model using human intestinal biopsies, demonstrating that chloride secretion induced by RV-NSP4 is oxidative stress-dependent and is inhibited by Sb, which produces soluble metabolites that prevent oxidative stress. The results of this study provide novel insights into RV-induced diarrhea and the efficacy of probiotics.

## Introduction

Rotavirus (RV) infection is the most frequent and severe form of acute gastroenteritis in infants and children worldwide and frequently requires hospitalization [1], [2]. Up to 40% of hospitalized children under 5 years of age with diarrhea are infected with RV [3], [4]. In developing regions, acute diarrhea still represents a leading cause of childhood mortality, second only to pneumonia, and RV is the most common agent [1], [5]. RV immunization has been identified as a major priority for the health of children by authoritative institutions [6]. No specific therapy is available, but selected probiotics, including *Saccharomyces boulardii* (Sb), reduce the severity and duration of diarrhea.

RV infects mature enterocytes of the small intestinal villi, inducing broad functional and structural damage [7]. In human enterocytes, RV diarrhea is the result of a sequence of combined secretory and osmotic mechanisms, including overstimulation of intestinal ion transepithelial secretion and intestinal damage, leading to malabsorption and osmotic diarrhea [8], [9]. Non-structural protein 4 (NSP4) plays a key role in secretory diarrhea. NSP4 is produced by RV and induces diarrhea in mice through the release of intracellular stores of calcium from enterocytes [10]. RV was recently shown to induce early, NSP4-dependent ion secretion [9], [11].

Redox imbalance is a common event in cells infected by viruses, but its role in RV diarrhea remains unclear. Oxidants, such as H_2_O_2_, induce anion secretion in selected segments of the intestinal tract, such as the rat ileum and colon [12], [13], and in an intestinal cell model [13], [14], but there is no evidence that oxidative stress induced by viral infections is linked with intestinal ion secretion. Redox imbalance is generally derived from a decrease in antioxidant enzyme levels, the depletion of cellular antioxidant defenses, and enhanced production of reactive oxygen species (ROS), leading to the rapid killing of infected cells and the release of viral particles [15]–[17]. A previous study reported that the oxidative/antioxidative profile is altered in gut homogenates from RV-infected mice, indicating oxidative stress [18]. In addition, RV induces a strong increase in mitochondrial superoxide dismutase expression [19]. Therefore, in this study, we investigated the involvement of oxidative stress in RV-induced diarrhea and the direct role of NSP4, if any.

Sb, a probiotic yeast, reduces diarrheal duration and the severity of RV gastroenteritis in children [20] and is recommended as an adjunct to oral rehydration solution by guidelines of authoritative institutions [21], [22].

*In vitro* and *in vivo* studies indicate that Sb exerts an antidiarrheal effect by acting on the resident microflora and inducing an anti-inflammatory effect [23]. The stimulation of brush border disaccharidases (e.g., lactase, sucrase) has been proposed as an additional mechanism to explain the antidiarrheal activity of this yeast [24]. None of these proposed mechanisms is consistent with the rapid efficacy observed in acute gastroenteritis, which is more consistent with a direct interaction of Sb with enterocytes and/or the virus than with modifications of intestinal microecology or immune regulation. It is becoming clear that several intestinal effects of probiotics are not associated with the direct interaction between the microorganisms and intestinal epithelial cells but are induced by soluble mediators released by the probiotics in the surrounding medium [25], [26]. The effects exerted on target cells by these released metabolic products have been designated the “postbiotic effect” [27]. Therefore, in the present study, we also investigated the effects of Sb-conditioned medium on RV-induced enterotoxic effects in our experimental model.

## Materials and Methods

### Intestinal Cell Line

Caco-2 cells were used as previously described [28]. Caco-2 cells were grown in Dulbecco’s modified Eagle minimum essential medium (DMEM; Life Technologies Italia, Monza, Italy) with a high glucose concentration (4.5 g/L) at 37°C in a 5% CO_2_ atmosphere. The medium was supplemented with 10% fetal bovine serum (FBS, Life Technologies Italia, Monza, Italy), 1% non-essential amino acids, penicillin (50 mU/mL), and streptomycin (50 µg/mL).

#### Virus strain and infection protocol

The simian rotavirus strain SA11 (RV) was used as previously described [9]. Briefly, the virus was activated with 20 µg/mL trypsin for 30 min at 37°C. The viral suspension was added to the apical side of cell monolayers. After 60 min, the cells were washed and incubated in FBS-free medium for the indicated time periods after infection.

### Purification of BacNSP4SA11

Sf9 cell monolayers (2×10^7^ cells) grown in Sf900 medium (Life Technologies Italia, Monza, Italy) in 175 cm^2^ flasks were infected with the recombinant baculoviruses BacNSP4SA11 (moi 10). When a cytopathic effect was observed, the recombinant protein was harvested from the cells lysed with lysis buffer (50 mM NaH_2_PO_4_, 10 mM imidazole, 300 mM NaCl, pH 8.0,, 1% Triton X-100, and 0.1% Protease Inhibitor Cocktail (Sigma-Aldrich S.r.l. Milan, Italy). The lysates were clarified by centrifugation at 22,000 g at 4°C for 5 min and purified by affinity chromatography using Ni-NTA agarose colums (Qiagen), following the manufacturer’s instructions. After 3 washes (with 50 mM NaH_2_PO_4_, 20 mM imidazole, 300 mM NaCl, pH 8.0), the His-tagged proteins were eluted in 400 µL of elution buffer (50 mM NaH_2_PO_4_, 250 mM imidazole, 300 mM NaCl, pH 8.0) and dialyzed against PBS. The purified 21–28 kDa HisNSP4 proteins, which corresponded to glycosylated NSP4, were visualized by SDS-PAGE and western blotting using a monoclonal anti poly-histidine antibody (Fig. S1). Protein concentration was quantified using the Bradford reagent (Bio-Rad, Milan, Italy) and several 0.2 mg/ml stock solutions were prepared.

An histidine-tagged HEV major ORF2 capsid protein of a swine hepatitis E virus (HEV) strain, expressed and purified as reported above for NSP4, was used as irrelevant control protein (Ruggeri F.M. unpublished). Then, we tested the effects of this protein in experiments on intestinal ion transport.

### ROS Production

ROS production was measured by 7′-dichlorofluorescein diacetate (DCFH-DA) spectrofluorometry. After stimulation, cells were exposed to 20 DCFH-DA (D6665; Sigma-Aldrich, St. Louis, MO for 30 minutes at 37°C in the dark. Intracellular ROS production was measured in a fluorometer (SFM 25; Kontron Instruments, Japan).

### DCF Fluorescence Imaging

Caco-2 cells were grown on glass cover slips for 3 days and were then fixed and permeabilized with paraformaldehyde (4%) and Triton (0.2%) for 30 min at 4°C. The cells were then incubated with 20 µM DCF-HA for 30 min at 37°C in the dark. Fluorescence images from multiple fields were obtained using a Nikon Eclipse e 80i microscope. The images were analyzed using NiS Elements D imaging software (Nikon Instruments Inc., NY, USA).

### GSH Assay

Intracellular levels of reduced (GSH) and oxidized glutathione (GSSG) were measured as described by Allen et al. [29] with a few modifications. Proteins were precipitated with 1% sulfosalicylic acid, and the supernatants were used to measure, in parallel, total and reduced glutathione. GSSG was determined by subtracting GSH from total glutathione. The GSH and GSSG contents were normalized for protein content and expressed as % of total glutathione.

### Ion Transport Studies

Ion transport experiments were performed in Ussing chambers (WPI, Sarasota, FL) as previously described [30]. Ion secretion was studied in Ussing chambers by monitoring increases in short-circuit current (Isc), as an indication of active, luminally directed anion secretion. Maximal changes in short circuit current (delta Isc) were recorded as an indicator of mucosal ion secretion. Neutralization experiments were performed using specific anti-NSP4 polyclonal antibodies. NSP4 (200 ng/ml) was incubated at 37°C for 1 hour with the antibodies (10 µg/ml) and then added to Caco-2 cells in Ussing chambers. The same concentration of preimmune antibodies was incubated with NSP4 and used as controls.

In experiments performed to investigate the role of Cl^−^ in the electrical response, Cl^−^ was substituted with SO_4_^−^ at an equimolar concentration. To investigate in greater detail the role of Cl^−^ in the electrical effect of NSP4, we used CaCCinh-A01 to inhibit TMEM16 channels [11]. Cells were incubated with CaCCinh-A01 (30 µmol/L), and electrical parameters were monitored. To investigate the role of Ca^2+^ in the effects of NSP4 Caco-2 cells were mounted in Ussing chambers with Ca^2+^ -free Ringer and NSP4 was added 30 min later. Parallel monolayers BAPTA-AM with Ca^2+^ -free Ringer alone or NSP4 served as controls.

### Transepithelial Resistance Measurement

The transepithelial resistance of cell monolayers grown on filters was measured using a Millicel-ERS resistance monitoring apparatus (Merck Millipore, Billerica, MA). The resistance was expressed in Ohms/cm^2^. Transepithelial resistance was measured at 24, 48, and 72 h after the specific stimulations.

### Preparation of Sb Culture Supernatant

Lyophilized Sb (Biocodex, Gentilly, France) was cultured in RPMI 1640 cell culture medium (100 mg/mL) for 24 h at 37°C. The cell-free culture supernatant (SbS) was obtained by centrifugation and passage of the Sb culture through a 0.22-mm filter. All studies were performed using SbS directly on Caco-2 cells.

### Human Intestinal Organ Culture

Biopsies from the distal part of the duodenum were obtained from 2 children seen at the Department of Pediatrics who underwent endoscopy for intestinal disorders. All biopsies were from macroscopically normal areas, and intestinal histology was subsequently reported to be normal. Organ culture was performed in DMEM with a high glucose concentration (4.5 g/L) supplemented with 0.5% FCS, 1% non-essential amino acids, 2% penicillin (50 mU/mL), and streptomycin (50 mg/mL) and incubated in 5% CO_2_/95% air for 1 h before treatment. Experiments were performed by adding RV (50 pfu/5 mm^2^) for 2 h to maximize the effect before spontaneous tissue disruption. Specimens were exposed to RV alone or were preincubated with SbS (2 h) and then homogenized in lysis buffer 100 mM Tris-HCl pH 7.5, 300 mM NaCl, 2% NP40, 1% Na deoxycholic acid, 0.2% SDS, 100 µg/mL PMSF, 5 µg/mL aprotinin, 1 µg/mL leupeptin, 0.7 µg/mL pepstatin). The GSH/GSSG ratio was determined as described above for cells. The experiments with human specimens were conducted with the understanding and written consent of each child’s parents, and the study methodologies conformed to the standards set by the Declaration of Helsinki.

### Ethics Statement

The study protocol (2008-001349-24) was approved by the Ethics Committee of the School of Medicine, University of Naples “Federico II” Italy. A written informed consent was obtained, for each enrolled child from the parents.

## Results

### RV Induces Intestinal Epithelial Oxidative Stress and Impairs Antioxidant Defenses

**Figure 1:**
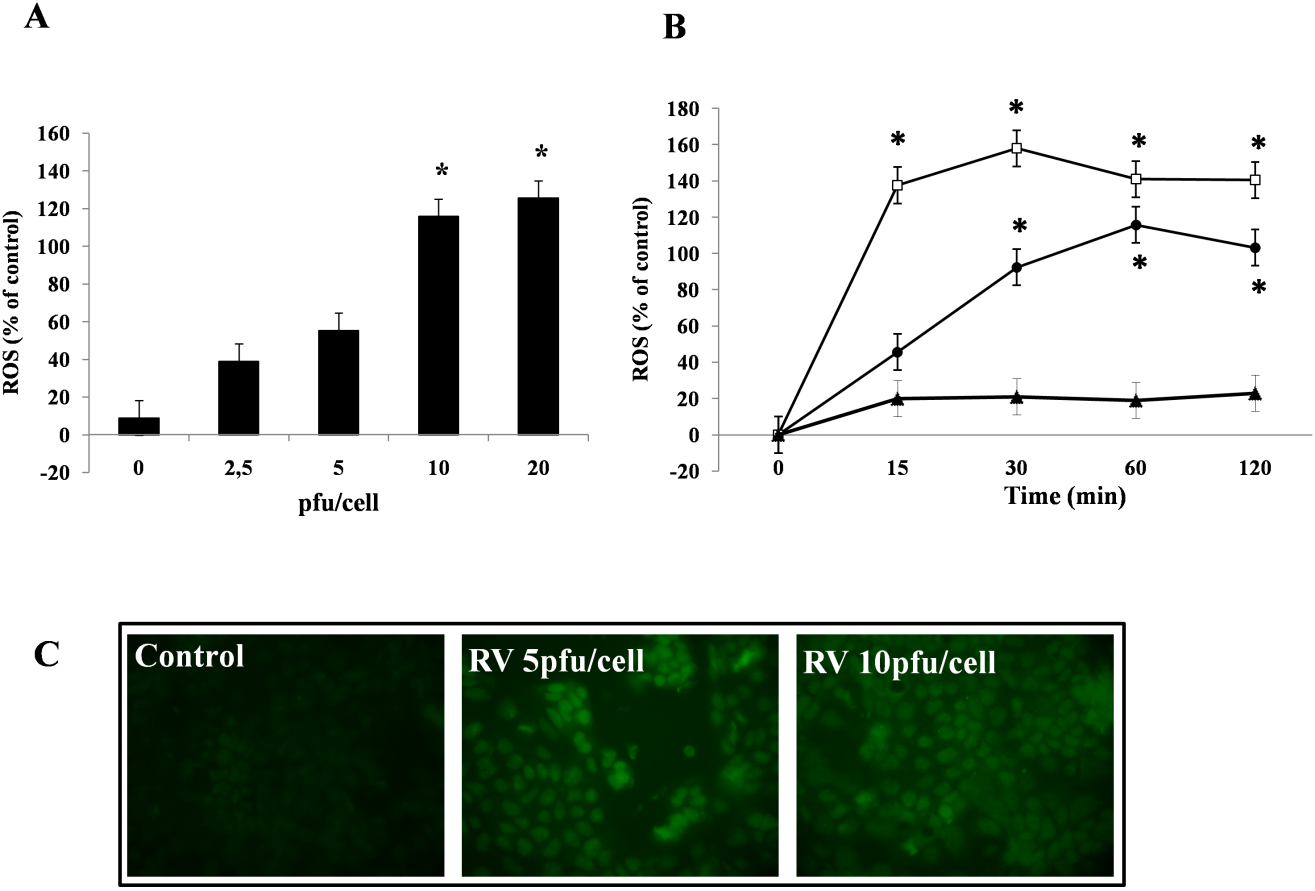
RV induces ROS generation in a dose- and time-dependent manner. Caco-2 cells were exposed to increasing dose of RV for 1 h (A) and to 10 pfu/cell for 15, 30 60 and 120 min post-infection (B). Intracellular ROS levels were evaluated by the DCFH-DA fluorometric method. RV (•), untreated cells as a negative control (▴), and H_2_O_2_ as a positive control (□). The data are representative of 3 separate experiments. *p<0.05 vs. 0 pfu/cell or time 0. (C) Immunofluorescent staining of ROS by DCFH-DA after 1 hour post-RV infection was compared with that in untreated cells (control). Representative staining is shown at 1 h post-exposure. Magnification: 200X.

To determine if RV alters the enterocyte oxidative state, we measured the intracellular levels of ROS and glutathione in Caco-2 cells. ROS levels progressively increased in cells exposed to increasing virus dose, with a maximal effect at 10–20 pfu/cell (Fig. 1A). Because ROS generation is usually rapid following a toxic stimulus, we performed time-course experiments in Caco-2 cells infected with RV for 15 up to 120 min. An increase in ROS was evident as early as 15 min after RV infection and reached its maximum level at 60 min (Fig. 1B). Intracellular ROS induction was confirmed by the increase in the green signal of DCF-DA by fluorescent microscopy in cells exposed to RV for 1 hour (Fig. 1C).

**Figure 2:**
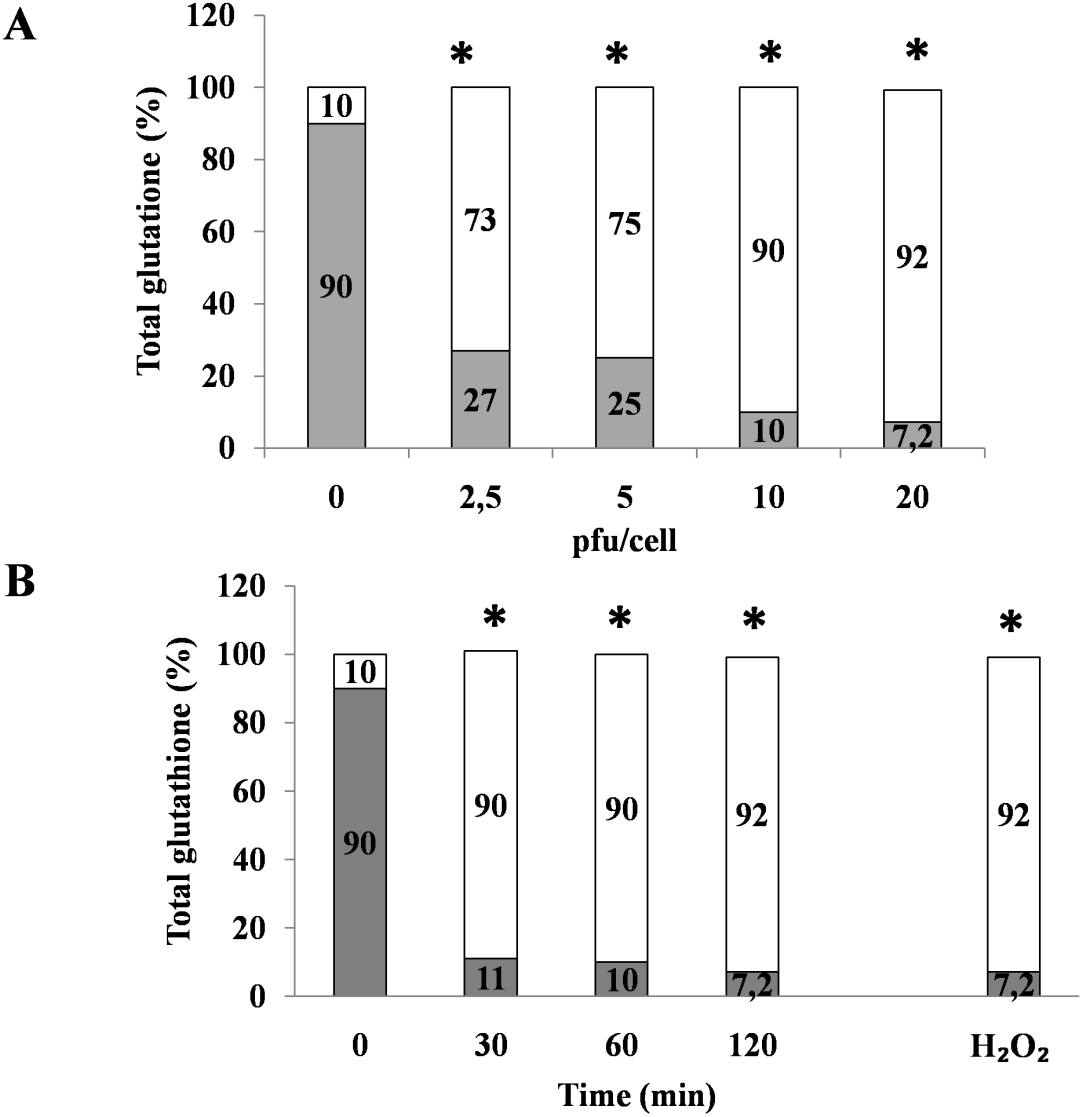
RV induces changes in intracellular antioxidant defenses. Caco-2 cells were exposed to different doses of RV for 1 h (A) and to 10 pfu/cell for 30, 60, and 120 min (B), and the ratio of GSH (grey) and GSSG (white) was evaluated. H_2_O_2_ was used as a positive control. the data are representative of 3 separate experiments. *p<0.05 vs. 0 pfu/cell or time 0.

We next investigated whether RV-induced ROS generation was associated with a decrease in antioxidant defenses by measuring glutathione, a major intracellular ROS scavenger. Glutathione protects cells against oxidative stress, and the intracellular proportions of GSH and GSSG are approximately 80−90% GSH and 10−20% GSSG under in uninfected cells. The GSH/GSSG ratio was reversed in RV-infected Caco-2 cells: 10% GSH and 90% GSSG. The effect peaked at 10–20 pfu/cell and was already evident as early as 15 min after infection (Fig. 2A and B).

**Figure 3:**
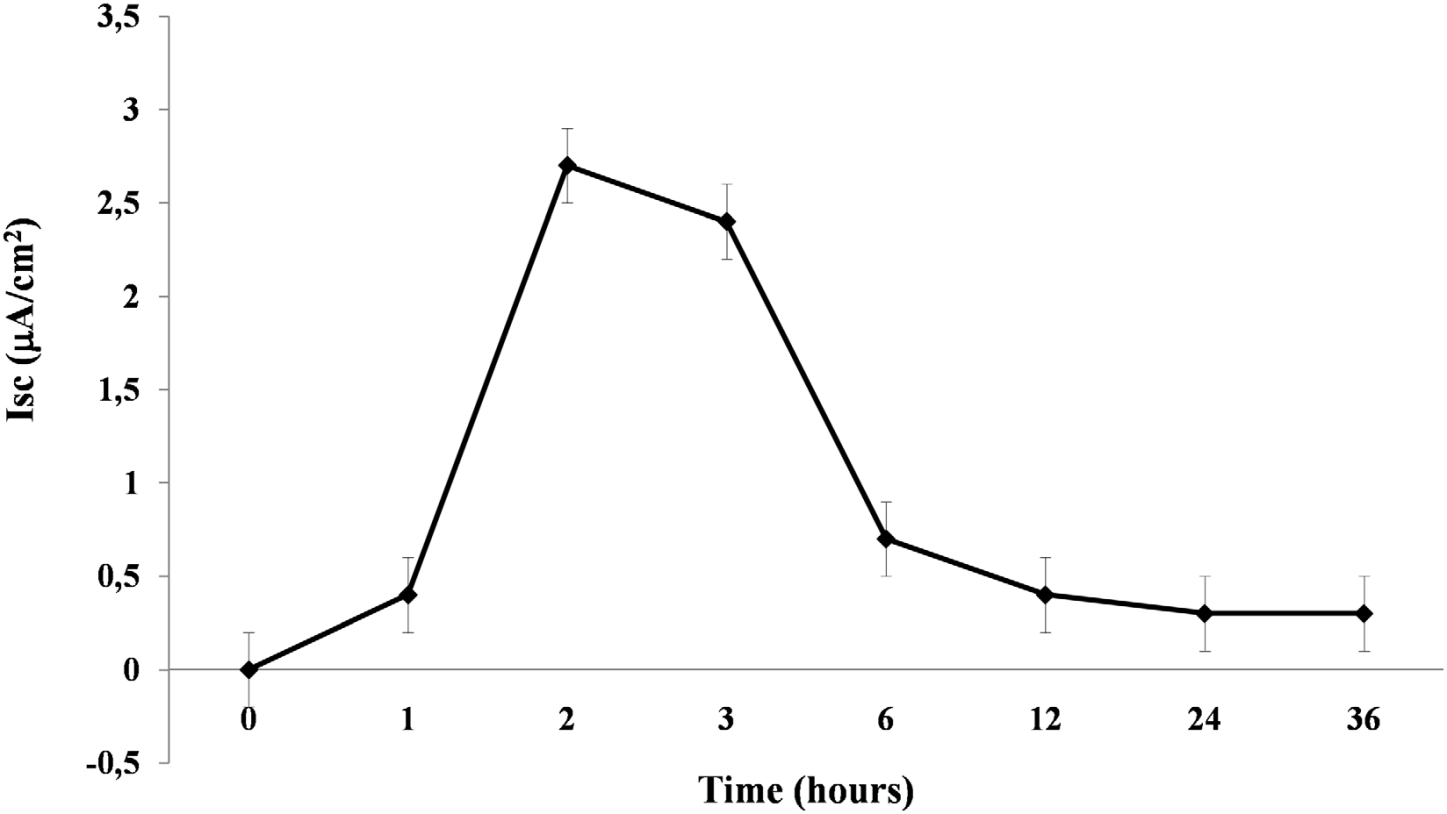
Rotavirus infection induces early chloride secretion. Caco-2 cell monolayers were infected with RV at 10 pfu/cell, and the Isc was evaluated in Ussing chambers. The data are representative of 3 separate experiments. *p<0.05 vs. time 0.

The addition of RV to Caco-2 cell monolayers resulted in an increase in the short circuit current (Isc) consistent with anion secretion (Fig. 3). The increase in the Isc was statistically significant at 1 h after infection, reached a peak after 2 h, and then slowly decreased. At 12 h after infection, electrical evidence of active ion secretion was no longer detected (Fig. 3).

### NSP4 Induces an Enterotoxic but not a Cytotoxic Effect in Caco-2 Cells

**Figure 4:**
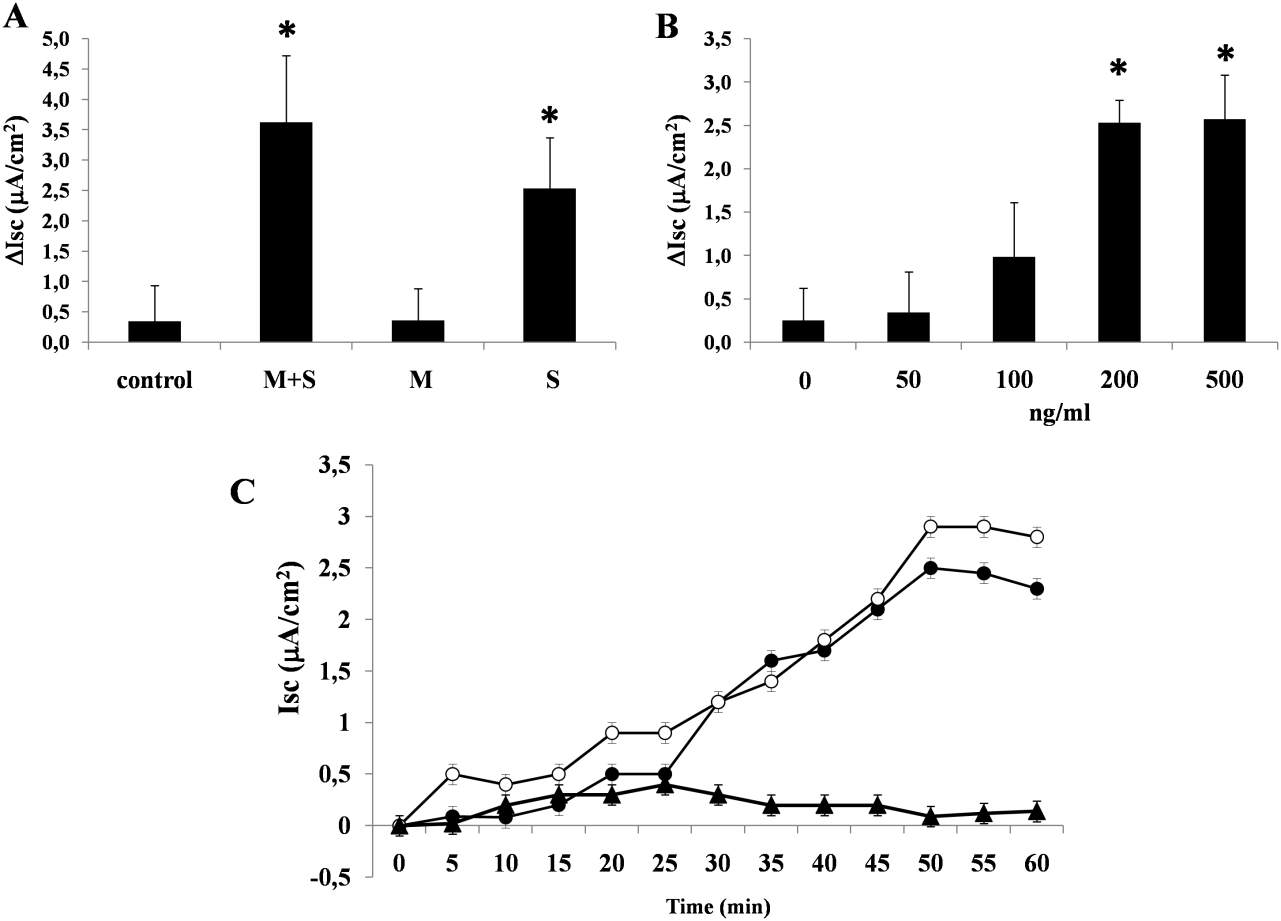
NSP4 induces chloride secretion in intestinal epithelial cells. (A) NSP4 (200 ng/mL) was added to the mucosal (M) or serosal (S) side or both (M+S) of Caco-2 cell monolayers for 1 hour, and the Isc was measured to evaluate chloride secretion. The maximal Isc shown was measured at 50 min time point. (B) NSP4 induced an increase in the Isc in a dose-dependent manner. The maximal Isc shown was measured at 50 min time point. (C) Caco-2 cells were infected with RV 10 pfu/cell (○) or exposed to NSP4 at 200 ng/ml (•) and Isc was measured for 1 hours every 5 minutes. A Isc similar increase was observed in RV infected cells and in virus-free cells exposed to NSP4. An histidine-tagged HEV ORF2 capsid protein was used as negative control (▴). The data are representative of 3 separate experiments. *p<0.05 vs. control or 0 ng/mL.

**Figure 5:**
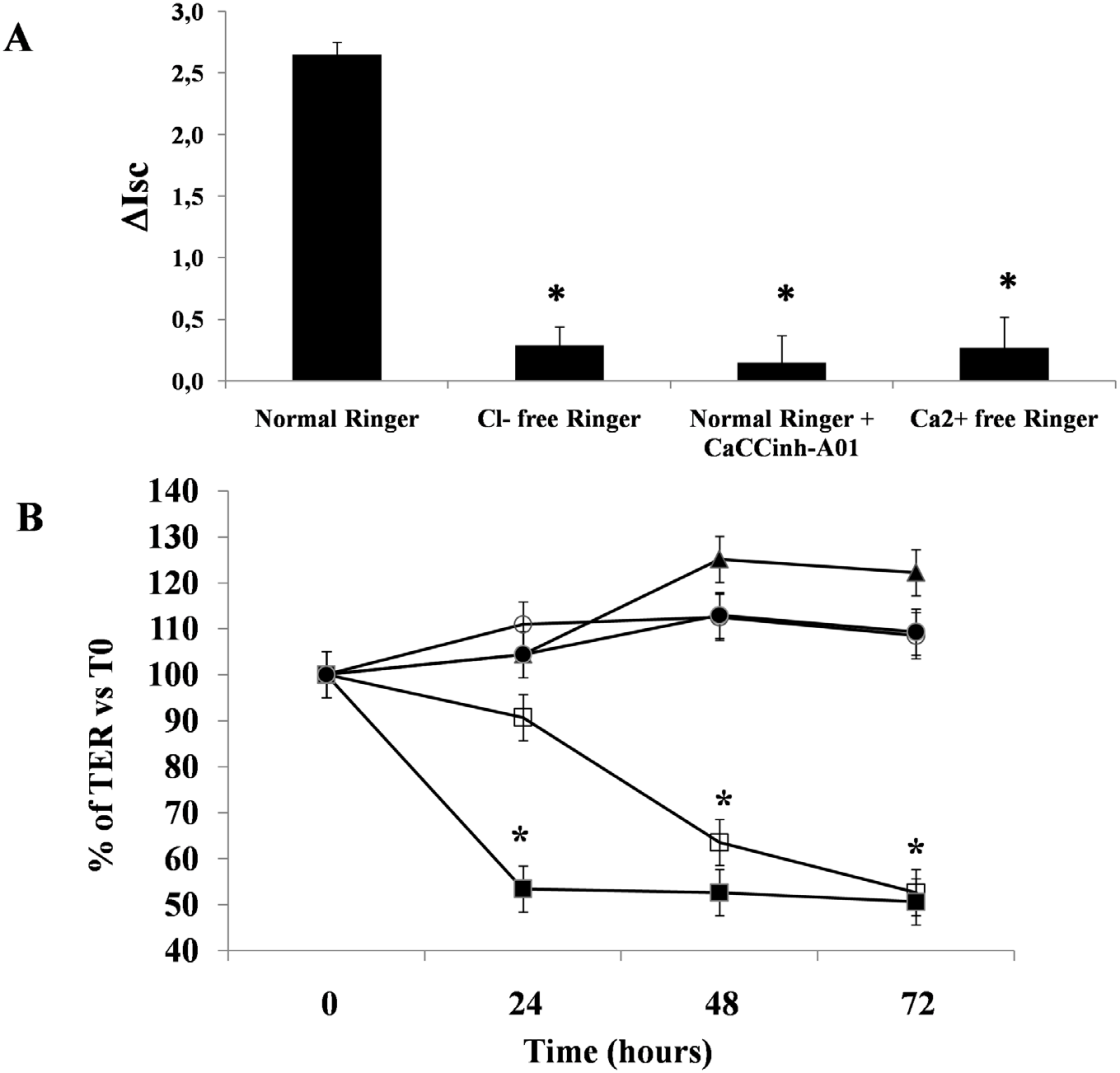
Modifications of Isc by NSP4 in various experimental conditions. (A) Changes in the Isc induced by pure NSP4 under various experimental conditions. The Isc was measured after the addition of NSP4 (200 ng/ml) in normal Ringer’s solution, chloride-free Ringer’s solution, Ringer’s solution supplemented with CaCCinh-A01 or Ca^2+^ free Ringer. Isc changes were measured after 50 min of stimulation. The data are representative of 3 separate experiments. *p<0.05 vs. normal Ringer’s solution. (B) The effect of NSP4 on intestinal epithelial integrity. The cytotoxic effect of NSP4 was evaluated by measuring TEER in Caco-2 cells. Cell monolayers were exposed to NSP4 at the serosal (•) or mucosal (○) side, to RV (□) and H_2_O_2_ (▪) as positive controls, or to vehicle as a negative control (▴). The data are representative of 3 separate experiments. *p<0.05 vs. time 0.

Because we previously observed that antibodies against NSP4 effectively inhibited the enterotoxic but not the cytotoxic effect of RV [9], we exposed Caco-2 cells to pure NSP4. NSP4 induced a significant increase in the Isc in the Ussing chamber experiments, consistent with electrogenic fluid secretion in Caco-2 cell monolayers (Fig. 4). The effect was dose-dependent and was observed when the viral protein was added to the serosal but not the mucosal side of the Caco-2 cell monolayers (Fig. 4A and B). The enterotoxic effect was evident as early as 30 min after the addition of purified NSP4 and reached a peak at approximately 50 min, after which the Isc value remained constant for 10–15 min (Fig. 4C). The pattern of the effect was similar to that previously observed in cells exposed to supernatants of RV-infected enterocytes [9]. To determine whether the enterotoxic effect was specific, we preincubated NSP4 with specific antibodies and then added the solution to Caco-2 cells in Ussing chambers. Specific antibodies significantly inhibited the electrical effect of NSP4 (NSP4 2,57±0,31 vs NSP4 with Ab 0,74±0,42; p<0.05). Incubation with preimmune antibodies had no effect on NSP4-induced increase in Isc (data not shown).To determine whether the electrical effect was caused by anion secretion rather than cation absorption, we performed the same experiments using Cl^–^free Ringer’s solution. In the absence of Cl^−^, the electrical effect was virtually abolished. Thus, the effect of NSP4 on the Isc was entirely due to transepithelial Cl^−^ secretion (Fig. 5A). We also added NSP4 at concentrations capable of eliciting the maximal secretory response (200 ng/mL) to Caco-2 cells in the presence of the TMEM16 channel inhibitor CaCCinh-A01. CaCCinh-A01 completely inhibited the secretory effect of NSP4 (Fig. 5A). To investigate the involvement of intracellular Ca^2+^ in the enterotoxic effects, cell monolayers were mounted in Ussing chambers with Ca^2+^ free-Ringer as described in the Materials and Methods. The subsequent addition of NSP4 resulted in a reduced increase in the Isc compared to NSP4 alone (Fig. 5A).

In our experimental model, NSP4 did not affect epithelial integrity as judged by TEER measurements. By contrast, TEER decreased in cells infected by RV (Fig. 5B).

**Figure 6:**
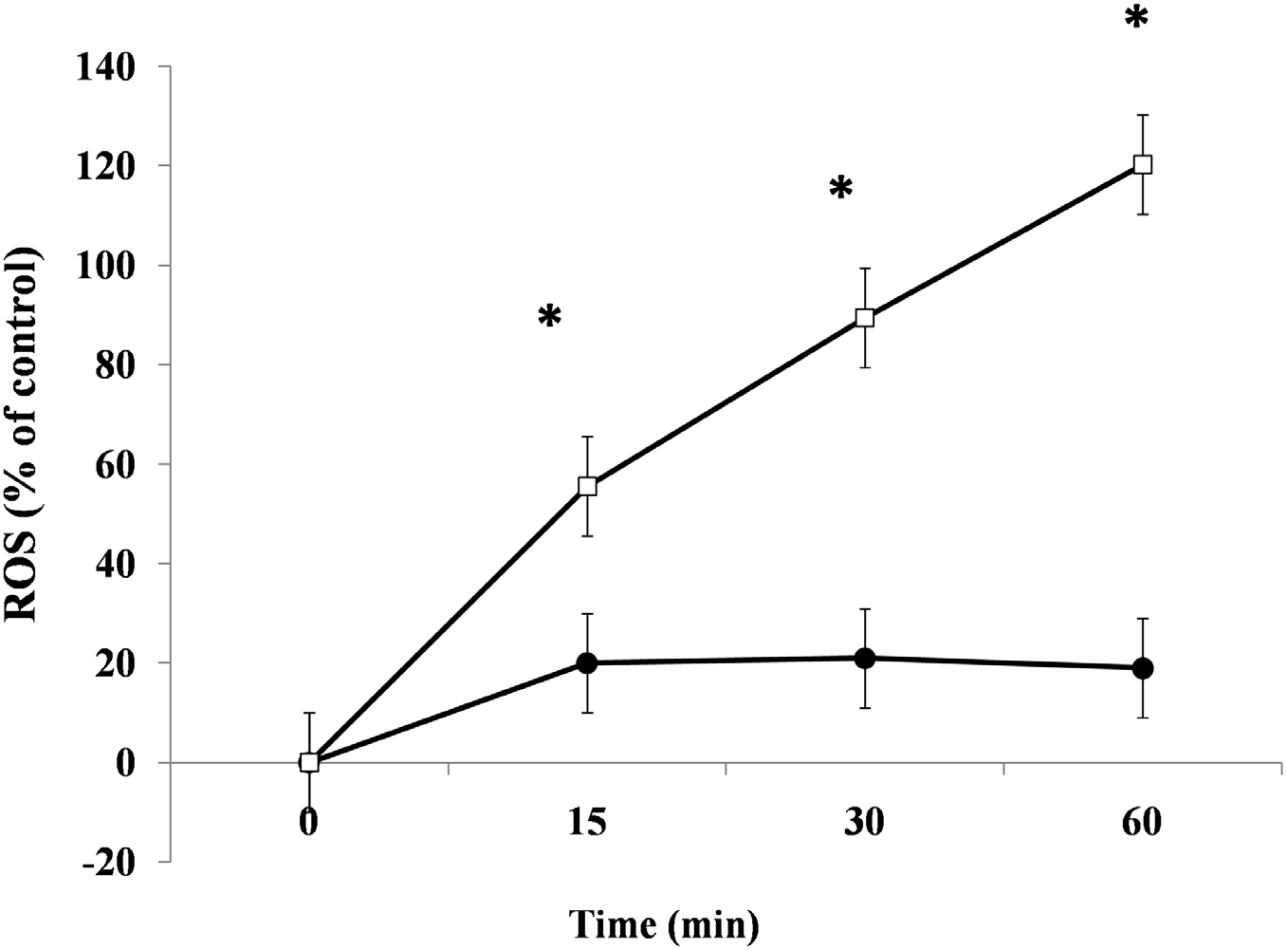
NSP4 induces time-dependent generation of ROS. Caco-2 cells were exposed to 200 ng/ml NSP4 (□) for 15, 30, and 60 min, and ROS intracellular levels were evaluated by the DCFH-DA fluorometric method and compared to a negative control (•). The data are representative of 3 separate experiments. *p<0.05 vs. time 0.

To determine if NSP4 induces oxidative stress, we stimulated Caco-2 cells with enterotoxin, and ROS levels were determined. As shown in Fig. 6, the addition of purified NSP4 induced ROS production in a time-dependent manner that virtually overlapped that observed for chloride secretion in Ussing chambers. These data demonstrate that the enterotoxic effect of RV diarrhea is directly and exclusively induced by NSP4 and is closely linked with ROS production.

### Oxidative Stress and Chloride Secretion Induced by RV and NSP4 are Strongly Inhibited by Pretreatment with Antioxidants

**Figure 7:**
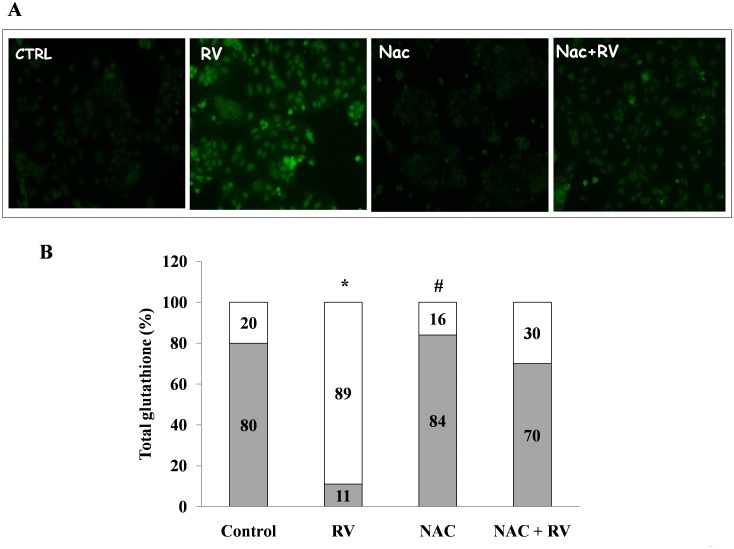
The effect of NAC on RV-induced oxidative stress in Caco-2 cells. (A) The addition of NAC (5 mM for 24 hours) to RV-infected cells completely inhibited ROS generation as shown by a representative staining at 1 h post-infection. Magnification: 200X. The data are representative of 3 separate experiments with 3–4 replicates for each experimental condition. (B) Effect of NAC (5 mM for 24 hours) on the RV-induced GSH/GSSG imbalance. The data are presented as the percent of GSH (grey) and GSSG (white) vs. total glutathione. The data are representative of 3 separate experiments. *p<0.05 vs. control; #p<0.05 vs. RV.

**Figure 8:**
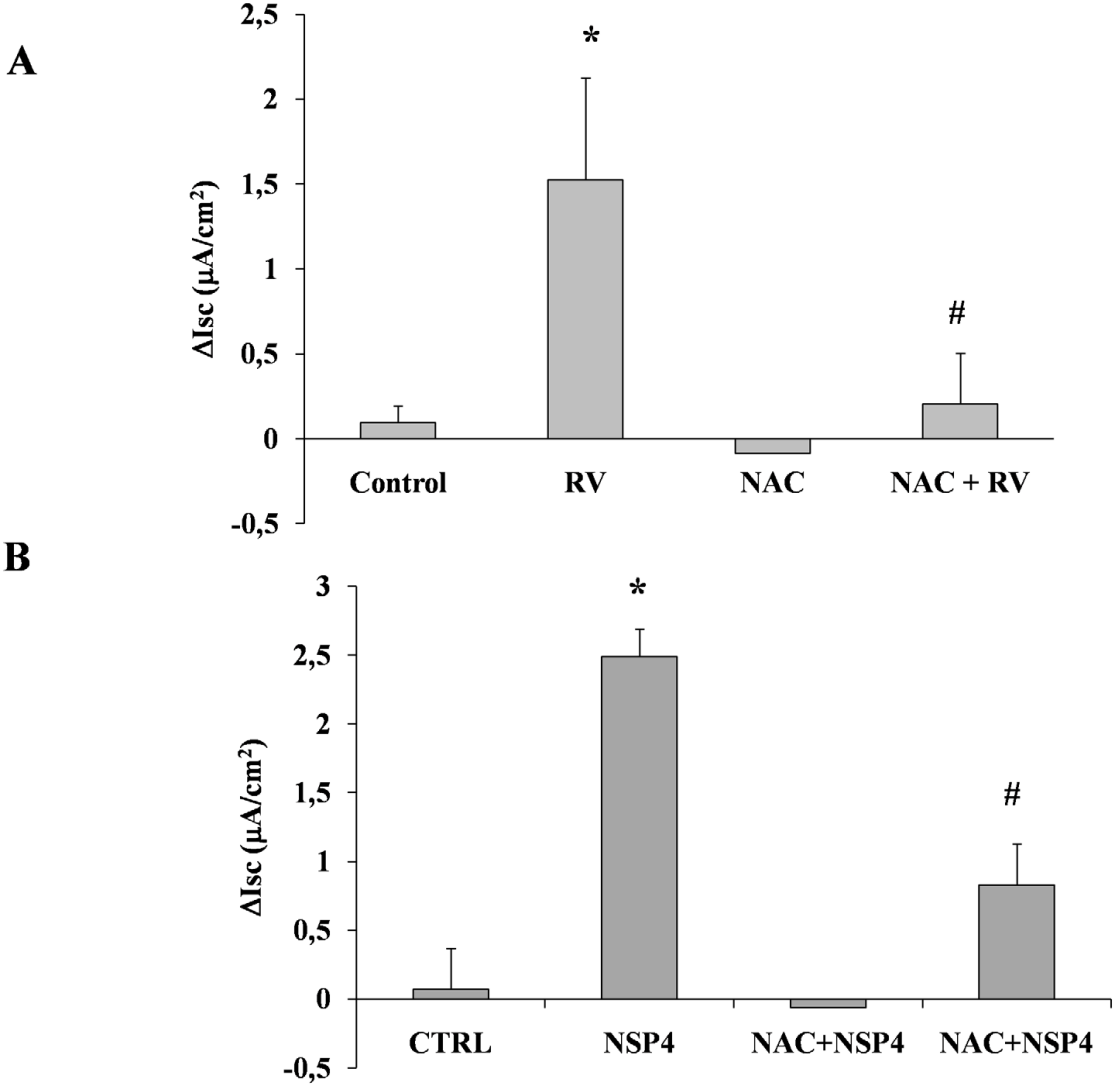
The effect of NAC on chloride secretion induced by RV and NSP4. (A) The addition of NAC (5 mM for 24 hours) to RV-infected cells completely inhibited the Isc induced by RV. (B) Pretreatment with NAC (5 mM for 24 hours) strongly inhibited the Isc increase induced by NSP4. The data are representative of 3 separate experiments. *p<0.05 vs. control; #p<0.05 vs. RV or NSP4.

To explore the relationship between oxidative stress and the enterotoxic effect induced by viral infection at the intestinal level, we preincubated Caco-2 cells with the antioxidant NAC. Pretreatment with NAC (5 mM for 24 hours) completely inhibited the RV-induced increase in ROS (Fig. 7A) and preserved the normal GSH/GSSG ratio (Fig. 7B). To further investigate the role of the redox imbalance induced by RV in chloride secretion, we performed experiments under conditions of oxidative stress prevention. Pretreatment with NAC (5 mM for 24 hours) completely prevented intestinal chloride secretion (Fig. 8A), suggesting that redox imbalance is a major mechanism in RV-induced secretory diarrhea.

To determine if oxidative stress is also involved in NSP4-induced chloride secretion, Caco-2 cells were pretreated with NAC and then stimulated with the viral enterotoxin. Under these conditions, the enterotoxic effect of NSP4 was strongly inhibited (Fig. 8B). NAC did not reduce the cAMP- or Ca^2+^ -mediated chloride secretion induced by Forkolin and Carbachol (Fig. S2 panel A) suggesting that NAC effect is not direct on these second messengers.

### SbS Prevents RV-induced Enterotoxic Effects and Oxidative Stress in Caco-2 Cells and Cultured Human Small Intestinal Mucosa

**Figure 9:**
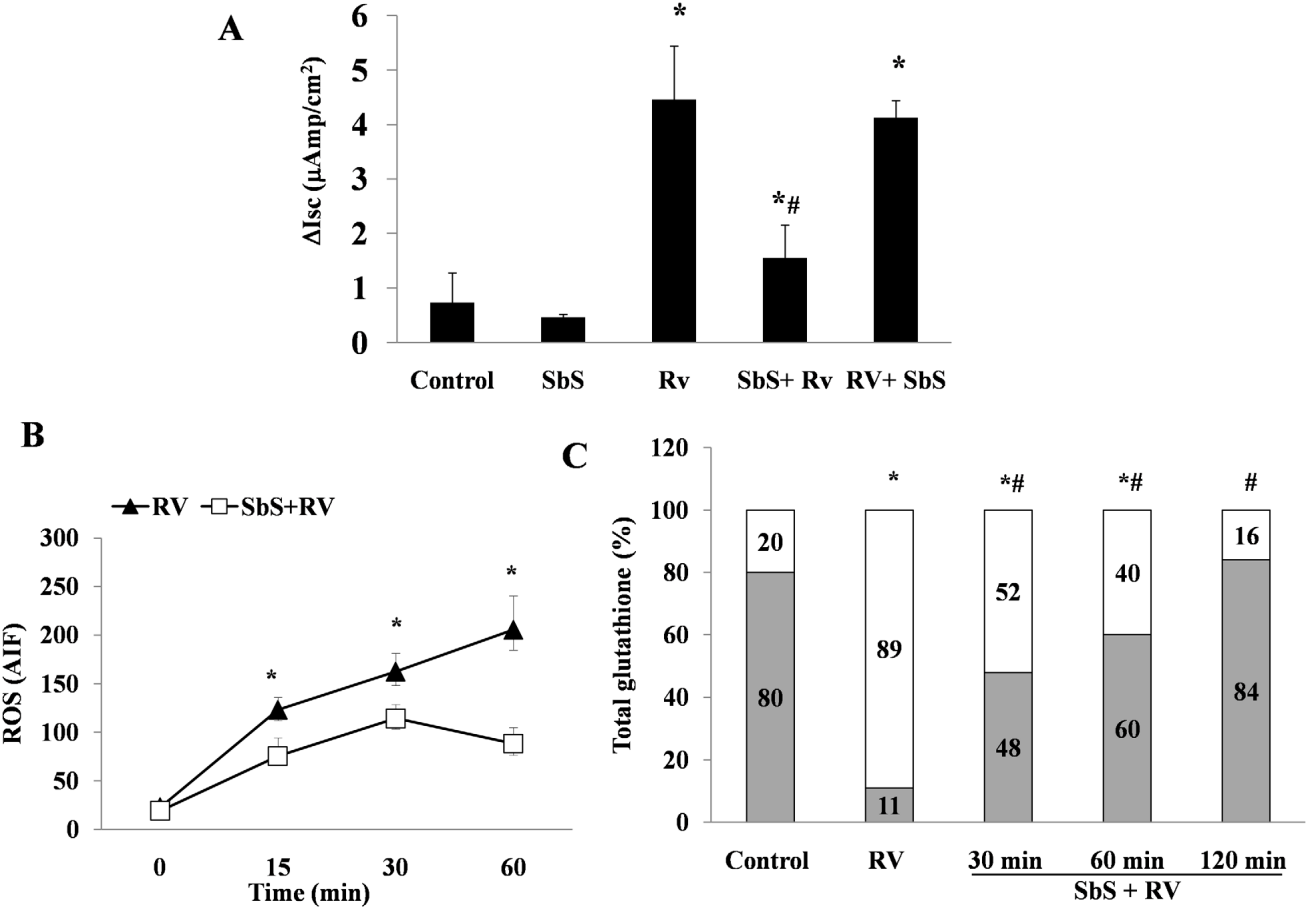
The effect of SbS on RV-induced chloride secretion and oxidative stress in Caco-2 cells. (A) The Isc, (B) ROS levels, and (C) the GSH/GSSG ratio were evaluated in RV-infected Caco-2 cells (10 pfu/cell) with (□) or without the addition of SbS (▴). The data are representative of 3 separate experiments. (A) *p<0.05 vs. control; #p<0.05 vs. RV. (B) *p<0.05 vs. SbS+RV. (C) *p<0.05 vs. control; #p<0.05 vs. RV.

To evaluate the effects of the probiotic Sb, which has been shown to be highly clinically effective, in our experimental model of RV-induced diarrhea *in vitro*, we added SbS to Caco-2 cells during the pre-infection phase and 2 h after RV infection (10 pfu/cell), then measured the Isc. SbS substantially reduced chloride secretion (Fig. 9A). This effect was observed when SbS was added before but not after virus infection. ROS levels and the GSH/GSSG ratio were evaluated in time-course experiments. The increase in ROS induced by RV was strongly inhibited in cells exposed to SbS compared to infected controls. The maximal effect was observed at 60 min (Fig. 9B). In addition, SbS reduced the GSH/GSSH imbalance at 30 min and restored the redox equilibrium to the same levels as in the control at 120 min after infection (Fig. 9C).

**Figure 10:**
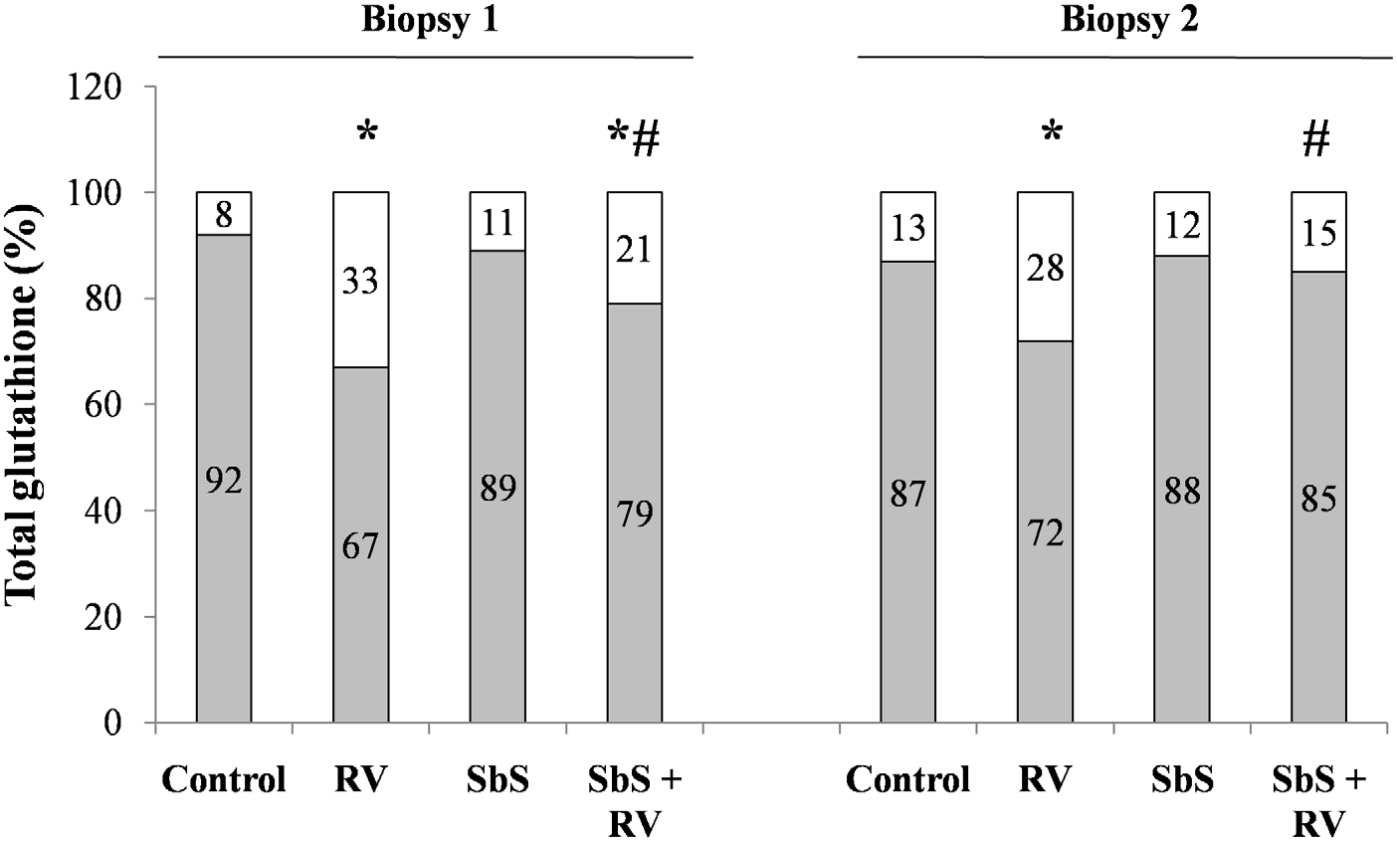
Antioxidant defenses in RV-infected human intestinal mucosa. Duodenal mucosal specimens were infected with RV (50 pfu/5 mm^2^) alone or in combination with SbS in an *ex vivo* organ culture model, and the GSH (grey)/GSSG (white) ratio was evaluated. *p<0.05 vs. control; #p<0.05 vs. RV.

Organ culture experiments were performed to compare the results obtained using Caco-2 cells with those in human tissue. Intestinal specimens were obtained from 2 children undergoing upper gastrointestinal endoscopy. After stimulation with RV (50 pfu/5 mm^2^) in the presence or absence of SbS, we evaluated the GSH/GSSG ratio. The GSH/GSSG ratio decreased upon RV exposure in intestinal biopsies exposed to RV for 1 h, confirming the oxidative stress pattern observed in Caco-2 cells. When SbS was preincubated for 30 min before RV infection, the ratio for both biopsies was similar to that observed in the controls, confirming that SbS prevented the GSH/GSSG imbalance induced by RV in human intestinal epithelia (Fig. 10).

Again, SbS did not reduce the cAMP- or Ca^2+^ -mediated chloride secretion induced by Forkolin and Carbachol (Fig. S2 panel B) suggesting that SbS effect is not direct on these second messengers.

## Discussion

NSP4 plays a substantial role in RV diarrhea. Since the first description of the NSP4 enterotoxin, a number of hypotheses have been proposed regarding its role in chloride secretion. The chloride secretory response is regulated by a phospholipase C-dependent calcium signaling pathway that is induced by NSP4 [31], and NSP4 plays a key role in ion secretion in human-derived enterocytes [9]. Ousingsawat et al. demonstrated that NSP4 modulates multiple pro-secretory pathways to induce diarrhea by activating the recently identified Ca^2+^ -activated Cl^−^ channel TMEM16A and inhibiting Na^+^ absorption by the epithelial Na^+^ channel ENaC and the Na^+^/glucose cotransporter SGLT1 [11]. We have now characterized the effects of NSP4 on ion secretion. The addition of NSP4 to Caco-2 cell monolayers resulted in the same electrical effect observed in Caco-2 cells infected with RV. Our results indicate that NSP4 exerts a polar effect in Caco-2 cells due to its interaction with the basolateral but not the apical cell membrane, suggesting that *in vivo* the viral protein acts when the epithelial integrity is damaged, thereby permitting contact of NSP4 with the basolateral side. It is possible that the decrease in short circuit current at later time points be due to disrupted tight junctions. However, the earlier secretion occur to be indeed directly by NSP4. In addition, the abrogation of the electrical response in the absence of Ca^2+^ or blocking TMEM16A channels, confirm the Ca^2+^ dependence as mechanism involved in the secretory effect. In addition, purified NSP4 induces ROS generation and GSH/GSSH imbalance with the same pattern as RV, further linking NSP4-induced oxidative stress to chloride secretion.

In gut homogenates of RV-infected mice, the oxidative/antioxidative profile is altered, indicating the presence of oxidative stress [18]. This effect was observed at a late stage of infection and might have been due to a decrease in glutathione recycling and/or production of glutathione-synthesizing enzymes. Our data provide clear evidence for a link between oxidative stress and RV-induced chloride secretion, which is the main mechanism of RV diarrhea.

Exogenous redox stressors induce chloride secretion depending on the site of action [32]. Our results demonstrate that the direct interaction between NSP4 and enterocytes leads to active chloride secretion, in agreement with a previous study in which intraperitoneal injection of NSP4 induced diarrhea in mouse pups [33]. Morris et al. demonstrated that the RV nonstructural glycoprotein NSP4 acts as a viral enterotoxin, inducing Ca^2+^ -dependent Cl^−^ secretion through Ca^2+^ release from intracellular stores in mice [33].

Our results provide further compelling evidence for this mechanism in human enterocytes. A previous study reported that infected Caco-2 cells maintain redox balance during RV infection [19]. The authors concluded that cell destruction caused by RV was likely not associated with oxidative damage to cellular components [19], suggesting that RV infection does not induce oxidative stress, enabling the accumulation of viral particles before cell destruction and virus release. The main difference with our results is in the timing of the observed effects, the sequence of which was clearly described in our original experimental model [9]. In particular, Gac et al. [19] evaluated oxidative stress at late time points post-infection, such as 48 and 72 h, whereas our findings indicate that RV induces an early increase in ROS production and a decrease in the GSH/GSSG ratio that is already detectable in the first hours following virus entry, suggesting that oxidative stress is a very early event.

There is consistent evidence that specific probiotic strains reduce the duration of RV diarrhea. However, the mechanisms of action of these probiotics are still unclear. Changes in the global structure of intestinal microflora, support of intestinal barrier function, stimulation of the immune response, and a number of other mechanisms have all been claimed as explanations of the efficacy against gastroenteritis. Sb has been shown to be highly effective against RV diarrhea in clinical trials [34], [35]. In our RV experimental model, SbS prevented RV-induced ROS production, increased antioxidant defenses, and reduced chloride secretion. The effect was observed using yeast-conditioned medium, suggesting that factor(s) secreted by the yeast were active in our system and induced a direct antisecretory effect, illustrating the so-called postbiotic effect of probiotics [36]. Sb-secreted factors were previously reported to be effective in the inhibition of proinflammatory cytokines [23]. In our experimental model, Sb inhibited RV-induced chloride secretion as a consequence of oxidative stress. A direct action on the enterocyte, with direct evidence of a consistent reduction of chloride flux from the serosal to luminal side, is in agreement with the rapid efficacy of Sb against diarrhea [20]. It is, therefore, a logical hypothesis that the protective effect against oxidative stress is the main mechanism underlying the clinical efficacy of Sb.

In conclusion, using a validated model of RV infection in human enterocytes, we demonstrated for the first time that RV induces chloride secretion through the generation of ROS, which a direct effect of NSP4. In addition, we determined that the supernatant of a culture of Sb acts on the glutathione-based defense system to limit chloride secretion. These results, which were obtained in an *in vitro* model of human-derived enterocytes and were replicated in human tissue, show a direct link between viral infection and the generation of oxidative stress, opening novel strategies to inhibit watery diarrhea induced by RV. These data also provide a new explanation for the high efficacy of Sb against childhood diarrhea observed in clinical trials. Specifically, taken together, these results demonstrate that the chloride secretion induced by the RV protein NSP4 is oxidative stress-dependent and inhibited by the postbiotic effect of Sb in human enterocytes.

## Supporting Information

Figure S1 **Purification of NSP4.** A) Western blot analysis of Sf9 infected with the recombinant baculoviruses BacNSP4SA11. NSP4SA11 (a) were observed as different glycosylated states (21–28 kDa) or the dimeric protein (50 kDa). Uninfected Sf9 cells were used as a negative control (b). B) Purification of BacNSP4SA11: (Ft) eluate, (W1/W2) washing buffer, (E1, E2, E3, E4) eluate fractions. C) SDS-PAGE analysis followed by Coomassie staining of NSP4SA11 protein purified from SF9 infected cells with the recombinant baculoviruses BacNSP4SA11 (+). SF9 uninfected cell lysates are also shown as control (−).
(TIF)

Figure S2 **Control experiments.** A) Caco-2 cells were preincubated with NAC and then stimulated with Theofilline (5 mM) or Carbachol (1 µM) and Isc was measured in Ussing chambers. B) Caco-2 cells were preincubated with SbS and then stimulated with Theofilline (5 mM) or Carbachol (1 µM) and Isc was measured in Ussing chambers. *p<0.05 vs CTRL.
(TIF)
