# Supplementary figures and images for: Chloride Secretion Induced by Rotavirus Is Oxidative Stress-Dependent and Inhibited by Saccharomyces boulardii in Human Enterocytes
Source: PLoS One. 2014 Jun 11;9(6):e99830. doi: 10.1371/journal.pone.0099830 (PMC4053528; doi:10.1371/journal.pone.0099830)

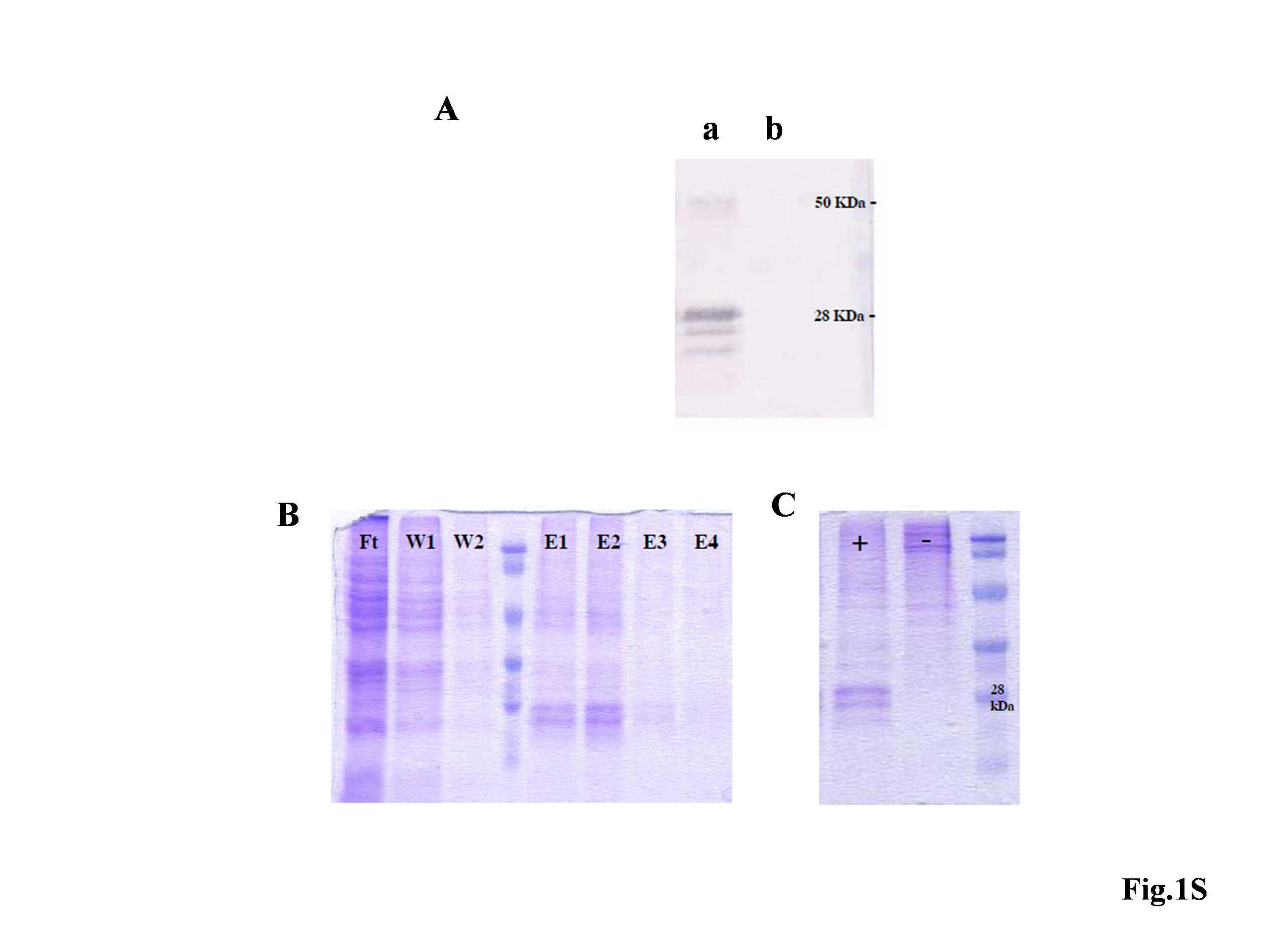

Supplement: Figure S1 — Purification of NSP4. A) Western blot analysis of Sf9 infected with the recombinant baculoviruses BacNSP4SA11. NSP4SA11 (a) were observed as different glycosylated states (21–28 kDa) or the dimeric protein (50 kDa). Uninfected Sf9 cells were used as a negative control (b). B) Purification of BacNSP4SA11: (Ft) eluate, (W1/W2) washing buffer, (E1, E2, E3, E4) eluate fractions. C) SDS-PAGE analysis followed by Coomassie staining of NSP4SA11 protein purified from SF9 infected cells with the recombinant baculoviruses BacNSP4SA11 (+). SF9 uninfected cell lysates are also shown as control (−). (TIF) [file pone.0099830.s001.tif]

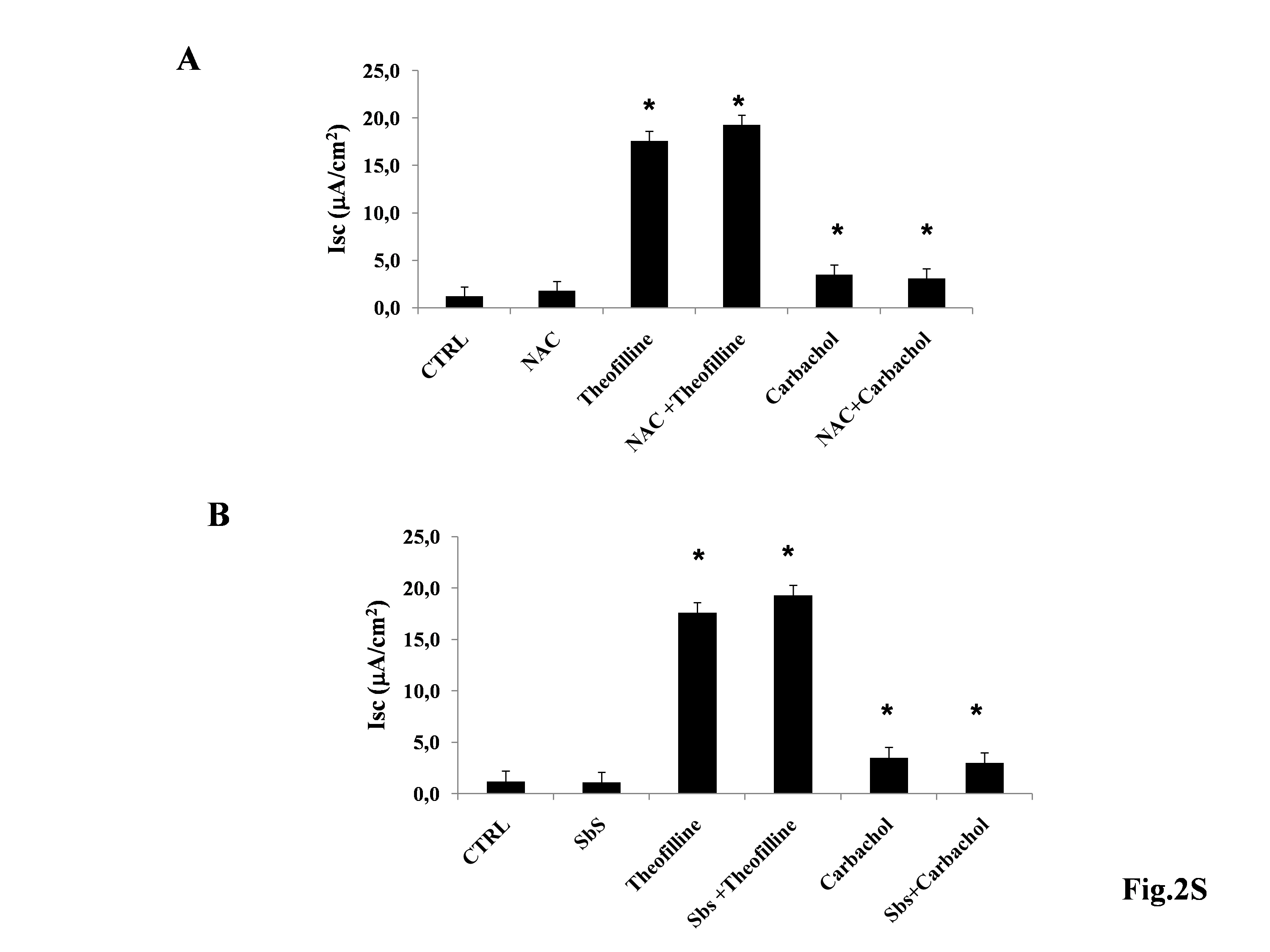

Supplement: Figure S2 — Control experiments. A) Caco-2 cells were preincubated with NAC and then stimulated with Theofilline (5 mM) or Carbachol (1 µM) and Isc was measured in Ussing chambers. B) Caco-2 cells were preincubated with SbS and then stimulated with Theofilline (5 mM) or Carbachol (1 µM) and Isc was measured in Ussing chambers. *p<0.05 vs CTRL. (TIF) [file pone.0099830.s002.tif]
